# The Analysis of Materials Strength Used in the Construction of the Flexible Underwater Bell—Batychron

**DOI:** 10.3390/ma15217768

**Published:** 2022-11-03

**Authors:** Grzegorz Rutkowski, Paweł Kołakowski, Katarzyna Panasiuk

**Affiliations:** 1 Faculty of Navigation, Gdynia Maritime University, 81-225 Gdynia, Poland; 2 Faculty of Engineering, Gdynia Maritime University, 81-225 Gdynia, Poland

**Keywords:** Batychron, flexible diving bell, thermoplastic polyurethane, underwater transport, hydro-technical device, tensile test, joining materials method

## Abstract

Batychron is a flexible underwater bell patented by the Gdynia Maritime University as a device used in hydro-technics engineering for underwater transport and diving while maintaining the safety of human life. This study aims to present the methods and results of strength tests and the conducted analysis of the selection of the most appropriate method of joining thermoplastic polyurethane film (TPU) and polypropylene belts for underwater use to obtain a device with a specific buoyancy force. A universal testing machine with a hydraulic drive was used for the tests. Various methods of joining polypropylene belts were tested to select the most favourable in terms of strength properties. For this purpose, two types of materials were selected: the TE324 polyester belt and the TS501_50 style belt. Various connection methods have been used: without seams; zig-zag stitch, straight cross; cross stitch, straight longitudinal; cross stitch, straight transverse, in order to select a joint with the highest strength parameters. In addition, the tensile strength of individual types of belts was tested. The methods of joining the TPU film were verified. The obtained results allowed us to determine that the strongest bond of TE324 material is a straight, longitudinal cross stitch. This is related to the load distribution in the belts tested in laboratory conditions, but also reflected in their practical application. Thanks to the results obtained, it was possible to select the optimal methods of joining (connection) and the construction of Batychron.

## 1. Introduction

Batychron is a flexible underwater bell patented by the Gdynia Maritime University as a device applicable in hydro-technics for underwater transport and diving while maintaining the safety of human life [1]. Diving bells are known for centuries [2,3], and there are several solutions for diving bells in hydro-engineering [4]. However, the disadvantage of the known solutions is the uniform mass construction of the bell and weight. These bells are always very heavy and need to be moved together with the vessel, requiring several people to be serviced. Under the assumption, Batychron should be light, handy, and portable. The construction of the canopy requires materials that are transparent, flexible, unbreakable when folded, and at the same time resistant to stretching, seawater, and UV rays.

**Figure 1:**
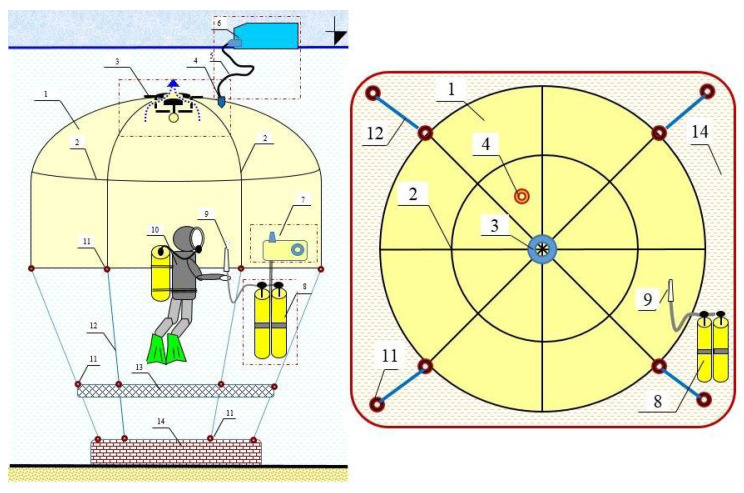
Project of Batychron—a flexible underwater bell: 1. Displacement module (dome); 2. Straps with a retaining net; 3. Measurement and control module with accessories; 4. Supporting module; 5. Flexible hose; 6. Breathing container; 7. The system that cleans the mixture of respiratory gases; 8. Breathing mix reservoir; 9. Breathing mix drain; 10. Diver; 11. “U” connector with a screw plug; 12. Liaison module with load, bearing belts; 13. Diving platform; 14. Anchor module. Source: Own research (2021).

The prototype of the device was made of Polyvinyl chloride (PVC) film, thermoplastic polyolefin (TPO) film and stylon-6 belts (TS501_50), which after several years of use in the marine environment under the influence of the sun and seawater have changed their colour to yellow, become dull and thus become less transparent and less durable. Materials showed high stiffness (especially at low water temperatures) and a relatively low safety factor against breaking and/or stretching of the Batychron’s structural elements. The research team from the Gdynia Maritime University decided to design and build a new upgraded version of the device named Batychron (Figure 1) with the highest quality materials available on the market these days. The project has been accepted in a grant program called “Innovation Incubator 4.0” implemented as part of the non-competitive project entitled “Support for the management of scientific research and commercialisation of R&D results in research units and enterprises” under the Intelligent Development Operational Program 2014–2020 (Measure 4.4). Pre-implementation work of UMG-03 (RWK/II4.0) [5].

**Figure 2:**
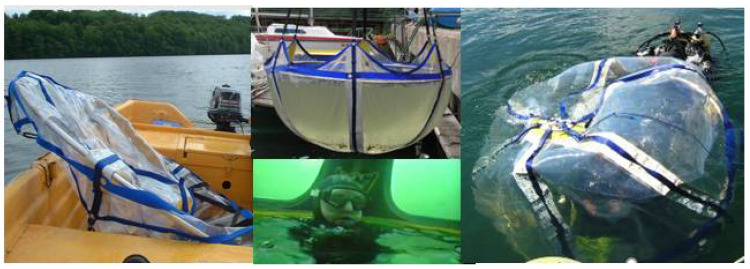
Strength water tests at Lake Radunskie. Source: Photos from own photos research (2022).

One of the goals of the above-mentioned project was to build a new device called the Batychron in a modular form. After research and material testing, the prototype was created. The utility model for this device was submitted to the Polish patent office (No. W.130766) on 5 May 2022. As a result of the research, it was proved that the device called Batychron can be used to secure and make all kinds of training, internship, tourist, recreational and sports dives more attractive. The installation of the Batychron, in particular, can be used to move sunken objects and carry out (in a controlled manner) the decompression of divers underwater, and save the lives of divers in emergencies—underwater base or the so-called underwater house for divers. Such a device could help to conduct underwater research [6] or rescue operations [7,8]. The pictures from underwater strength tests at Lake Radunskie has been shown in Figure 2.

From the research carried out, the width of the construction materials should be at least 100–120 cm and their thickness depend on the tensile strength of the materials (minimum unit load of 0.025 kg/cm^2^ for VI and VIII informative versions). Herein, during material market research, component materials for flexible underwater bell construction were selected thermoplastic polyurethane (TPU) film and TE324 polyester belts [5]. In underwater applications, thermoplastic polyurethane elastomers (TPU) are often used [9] Thermoplastic polyurethane elastomer (TPU) is used, e.g., as a housing in underwater sonar devices [10,11]. 

Thermoplastic polyurethane (TPU) is widely used in various fields, demonstrating excellent mechanical performance, corrosion resistance, ease of processability, and chemical stability [12,13,14]. The research in [15] verified the absorption and diffusion of TPU. The influence of aging on thermal, mechanical, and tribological properties was analysed. Aging had a direct effect on mechanical properties. The modulus of elasticity and the stress at 200% strain of the tested TPU decreased after sufficient exposure to moisture. On the other hand, the mechanical properties of the material surface were equally tested with the wear test. A decrease in wear resistance of aged TPU was found. This study also assessed the reversibility of mechanical and physical properties upon exposure to moisture. It has been found that the degradation of the polymer is an irreversible phenomenon. In the article [16] the influence of the artificial atmospheric environment on the characteristics of thermoplastic polyurethane (TPU) material was analysed. The appearance and thermal and mechanical properties of the material subjected to the aging process were verified. Changes in the appearance and morphology of TPU material after exposure to UV radiation were revealed with the use of optical microscopy and scanning electron microscopy (SEM) [17,18].

Identifying that the selected materials will be suitable as components for the construction of the underwater bell structure with a specific displacement, these materials need to be subjected to a series of strength and fatigue tests in various configurations of joining them together. There are many methods of joining materials, from standard methods, such as joining by needle stitching, to modern methods, such as ultrasonic methods [19]. 

Therefore, the objective of the herein study is to present the methods and results of strength and fatigue tests as well as the systematically investigated analysis for the selection of the most suitable way to combine TPU film and polypropylene belts to be used underwater for a device at a specific buoyancy force.

The remaining sections of the study are as follows. The materials and methods employed in answering the research questions mentioned in the current work are presented in Section 2. Section 3 presents the results and discussions. Conclusions, research gaps, and recommendations from the current study are highlighted in Section 4.

## 2. Materials and Methods

The assumption is that the method of joining the material should ensure tightness and adequate tensile strength in the displacement dome of the Batychron device. In this section, the authors presented materials for the construction of an underwater diving bell, various methods of joining individual components as well as strength and fatigue tests of these joints.

**Figure 3:**
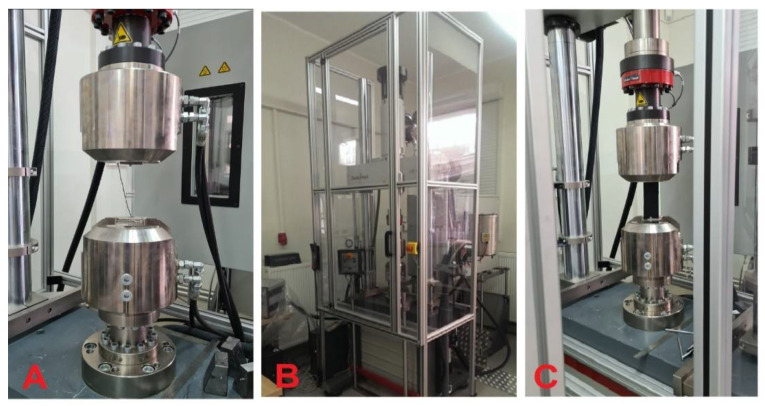
The stand for mechanical tests: (**A**) TPU Tensile strength test; (**B**) overall of the Zwick Roell universal testing machine for mechanical tests; (**C**) TE324 polyester belts strength test Source: Own research (2021).

The thermoplastic polyurethane as a construction material for a flexible underwater bell was verified based on the mechanical properties of this material presented in Table 1. The idea of the method [20] was to verify material strength, elasticity, and elongation at the break by the static tensile test on various ways of combining this material. The tests were carried out on the Zwick Roell universal testing machine and the TestExpert II software. Figure 3 shows a stand for mechanical tests. 

Strength tests were performed with TPU samples about 15 cm wide and 30 cm long, with a material bonding length of about 30 cm. Such samples were cut into narrow strips 2 to 3 cm wide and tested under various conditions. The strength test was obtained with 5 series of each joining method. In the case of joining, the glue with overlaps and the High-frequency welding method was used. High-frequency welding (often abbreviated as HF welding or RF welding) is a process in which plastics are joined together by means of an electromagnetic field. The obtained connection can be very strong—often close to the original strength of the joined materials. In some scenarios, the seam might be even stronger than the original materials [21,22]. The following joining methods have been used for tests:

- TPU film bonded with glue (overlap at 5 cm);
- TPU film bonded with glue + hardener (overlap at 5 cm);
- TPU film joined by HF Welding (microwave—5 cm overlap, 5 s, 3 bars).

**Figure 4:**
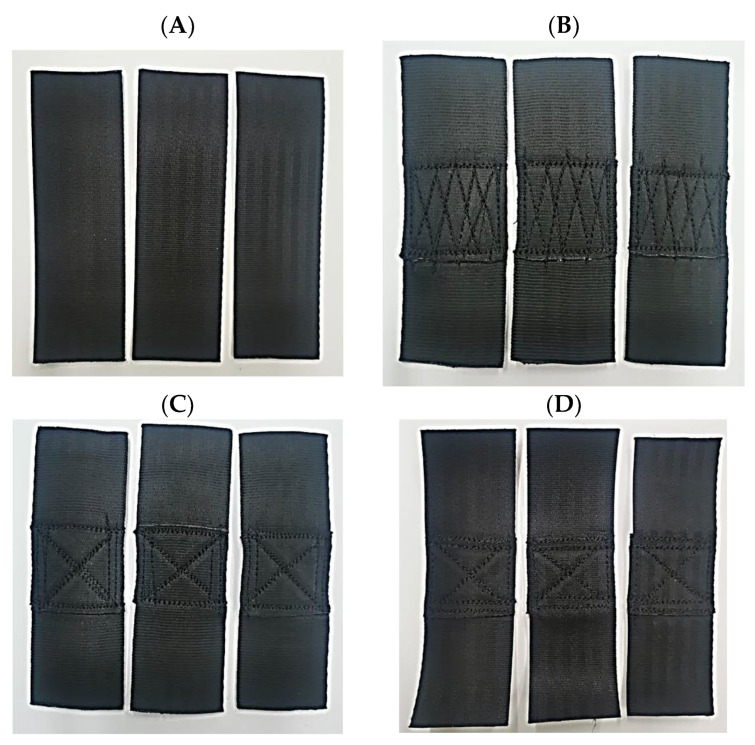
TE324 polyester belts: (**A**) without seams; (**B**) zigzag stitch, straight transverse; (**C**) straight, longitudinal cross stitch; (**D**) cross stitch, straight cross stitch; Source: Own research (2021).

**Figure 5:**
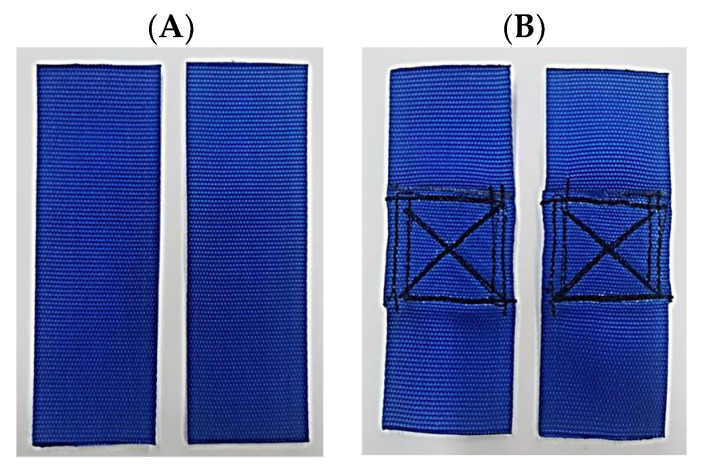
TS501_50 stylon belts: (**A**) without seams; (**B**) straight, longitudinal cross stitch; Source: Own research (2021).

The second component of the Batychron device is polyester belts—designed to connect the entire structure, and their connection. This is of importance in terms of ensuring the safety of the entire structure. Hence, two types of belts were verified and analysed: polyester belts and stylon belts (TE324 and TS501_50). Figure 4 presents the TE324 polyester belts and the types of joints. Since stylon-6 belts (TS501_50) were used in the earlier construction, it was decided to verify their properties and compare them. Figure 5 presents the stylon belts without joining and with a cross stitch. 

The strength of the belts and the tape fastening the openwork structure of the Batychron installation was tested. Ten samples of different belts, joined by different sewing methods, were subjected to strength tests, each sample having a length of 15 cm with an overlay of connected materials of 5 cm. The various methods of joining (stitching) belts were tested:

- Without seams;
- Zig-zag stitch, straight cross;
- Cross stitch, straight longitudinal;
- Cross stitch, straight transverse.

## 3. Results and Discussion

The strength test of TPU film was obtained with five series of each joining method. Due to the lack of the possessed instrumentation, the tests ended with an elongation of approx. 250%, during the test on each, mentioned joining methods. This material showed great flexibility, as after removing the given load, it returned to its original shape. As a result of the tests, it was decided that the new design of the Batychron will be made of TPU film with a wall thickness of 1 mm (TPU 1000 μm Clear type) with a strength of about 200 kg/cm2, which will be joined by HF Welding (microwave 5 cm overlap, 5 s, 3 bar).

The results of the belt’s static tensile test were obtained. It was noticed that most of the belts broke with a load of about 15,000 N, although they had strength certificates from the manufacturer for a strength of over 2 tons. The following parameters were obtained: tensile strength, Young’s modulus, and elongation. Table 2 presents a summary of the obtained results (average of five trials).

**Figure 6:**
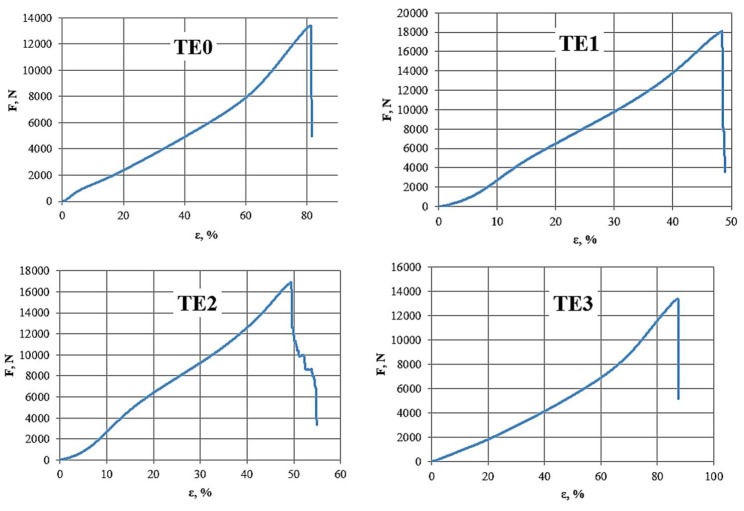
*F* (*ε*) diagrams of sample TE324 samples subjected to a static tensile test; Source: Own researches (2021).

Based on the results presented in Table 2 and the diagrams in Figure 6, it can be observed which types of joining are characterised by higher values of force, stress, deformation, and also the modulus of longitudinal elasticity.

The TE324 (TE0) material is characterised by a small value of the maximum force concerning the joined materials, although it exhibits the highest value of tensile strength and a relatively high value of deformation. In the case of the zigzag stitch (TE1), the highest values of force were obtained; however, in the case of tension, the values are approx. 30% lower—which could be expected. The deformation is also lower by approx. 33% compared to the non-connected belt. In this case, an increase in the longitudinal modulus by approx. 21% is favourable. For the cross stitch (TE2), favourable values of the maximum force were obtained, compared to the material without seams higher by approx. 26%. In the case of the tensile strength (*σ*, MPa), the obtained values were much higher than other types of joining (TE1, TE3). The deformation was comparable to the zigzag stitch, while the modulus of longitudinal elasticity is significantly higher than the others, about 75% higher than the belts without joining, and also about 43% higher than the zigzag stitch (TE1). In the case of joining the TE3 type belts, the lowest values were obtained in terms of maximum force, tension, and Young’s modulus, while the deformation was obtained at the level of approx. 90%, which is a much higher value compared to other materials. When it comes to the use of joining in the Batychron of TE324 material, the most preferred is a cross-stitch, straight longitudinal (TE2). It is characterised by the highest value of stress and Young’s modulus, the value of the maximum force is slightly smaller than the zigzag stitch, as is the deformation.

**Figure 7:**
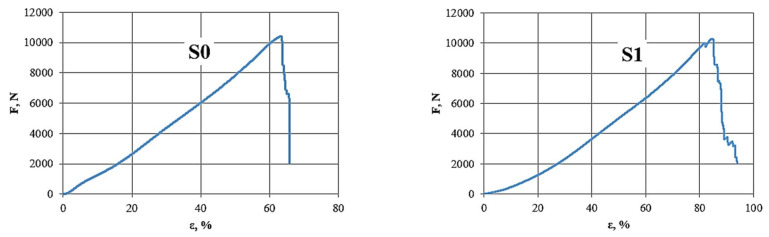

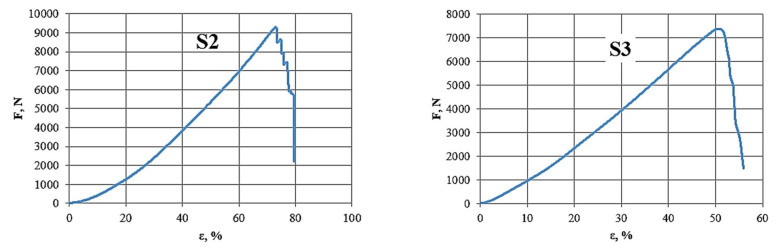
F (*ε*) diagrams of sample TS501_50 samples subjected to a static tensile test; Source: Own research (2021).

**Figure 8:**
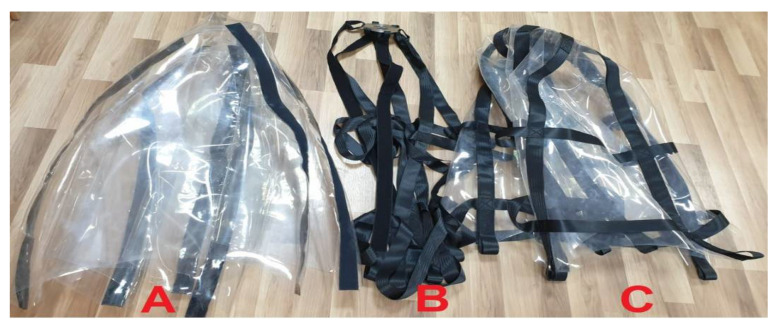
Batychron joining of main components: (**A**) transparent dome made of TPU material; (**B**) openwork mesh made of polyester belts; (**C**) Flexible diving bell—Batychron; Source: Own photos (2022).

The TS501_50 material, unlike the TE324 material, is characterised by a much lower maximum force when tensile. In the case of samples without joining, the maximum forces were obtained at the level of 9676 N, which is a value 28% lower than in the case of T0 samples (TE324 without joining), this material is weaker. By using a combination with certain types of stitching, much higher values are obtained. When using a zigzag stitch with straight transverse (S1), the maximum force is approx. 6% higher, but this is not reflected in the tensile strength, taking into account the sample dimensions, where it decreases by approx. 36%. The same goes for Young’s modulus. The use of a zigzag stitch with straight longitudinal (S2) and a cross stitch with straight transverse (S3) does not improve the strength properties. The only advantage of analysing the strength parameters of the TS501_50 material is its high deformability (*ε*, %) concerning the TE324 material. Figure 7 shows the *F* (*ε*) graphs of TS501_50 samples subjected to the static tensile test and a joining of the main components (transparent dome made of TPU material and openwork mesh made of polyester belts) has been shown in Figure 8.

## 4. Conclusions

The TPU film with a wall thickness of 1 mm (TPU 1000 μm Clear type) with a strength of about 200 kg/cm^2^ has been selected as the most appropriate material to construct the dome of the Batychron. For the TPU film joining method has been selected the High-Frequency Welding (microwave 5 cm overlap, 5 s, 3 bar) is the most appropriate for such a device. The first device has been made of Polyvinyl chloride (PVC) and thermoplastic polyolefin (TPO) films. These materials were weaker and less flexible than TPU. Moreover, the materials after several years of use in the marine environment under the influence of the sun and seawater become dull and thus become less transparent and less durable.

Based on the analysis of the obtained results, it was possible to select the appropriate connection of the belts, which are very important for the entire structure of the Batychron. After carrying out the strength tests of two materials TE324 and TS501_50, the obtained results were verified in detail in terms of the type of joining, which were used in the construction. From among the tested materials, the TE324 material was selected, characterised by significantly higher strength parameters than the TS501_50 material. In the case of splicing, various types of stitching were tested, such as the zigzag stitch with the straight cross stitch, the cross stitch with the straight cross stitch, and the cross stitch with the straight length, and compared with the fabrics without the joint. The obtained results allowed us to determine that the strongest bond of TE324 material is a straight, longitudinal cross stitch. This is related to the load distribution in the belts tested in laboratory conditions, but also reflected in their practical application.

In the first version of the Batychron, the joining of materials was made by sewing. The entire canopy consisting of PVC and TPO film and TS501_50 stylon stripes was made as one element. Such a structure was less flexible, it contained more interference with both materials, which made it less resistant to high displacement loads under the water.

In the new version two key components, i.e., an openwork mesh made of polyester belts and a transparent dome made of TPU material, are connected by velcro. The velcro was glued to the TPU film in the places where the material was welded together and sewn to the load-bearing mesh made of belts. Under the water, the Batychron dome press against the openwork mesh under the influence of the buoyancy force generated inside the underwater diving bell. This method prevents the need to sew these two components, which was an undesirable effect of the first version of the flexible underwater bell. Moreover, the modular method of new designed Batychron allows the replacement of any damaged part without the need to repair the entire device.

Summing up, it can be stated that the Batychron dome, made according to the above guidelines, meets the safety standards, which was also confirmed by sea tests carried out in lakes and the sea area of the Gulf of Gdansk. Strength tests and sea tests carried out in various water reservoirs (sea basins and lakes) and at various depths (from 5 to 35 m deep) also confirmed the high resistance of the construction materials used to the influence of changing hydrometeorological conditions in a given reservoir. The authors plan to continue this research. In the near future, samples of materials that are currently lying at the bottom of various research basins will be subjected to repeated strength tests. We plan to publish the results of future research in the same journal.

## Figures and Tables

**Table 1:** The mechanical properties of pure TPU.

x	Tensile Strength Rm [MPa]	Elongation at Break [%]	Elastic Modulus [MPa]
TPU	176	313	8.43

**Table 2:** Summary of the obtained results from the static tensile test; Source: Own research (2021).

Type of Material	Mark	*F*, N	*σ*, MPa	*ε*, %	*E*, MPa
TE324 without seams	TE0	13397	164	72	32
TE324 zigzag stitch, straight cross	TE1	17467	117	48	39
TE324 cross stitch, straight longitudinal	TE2	16929	121	49	56
TE324 cross stitch, straight transverse	TE3	13470	90	90	26
TS501_50 without seams	S0	9676	113	61	20
TS501_50 zigzag stitch, straight transverse	S1	10291	73	84	12
TS501_50 cross stitch, straight longitudinal	S2	9289	66	72	8
TS501_50 cross stitch, straight cross stitch	S3	7387	47	50	22

*F*—maximum force, N; *σ*—tensile strength, MPa; *ε*—strain, %; *E*—modulus of longitudinal elasticity (Young’s modulus), MPa.

## Data Availability

Not applicable.
